# Exploring the prospect of intrinsic wave propagation in evaluating myocardial stiffness among patients with type 2 diabetes

**DOI:** 10.3389/fcvm.2023.1162500

**Published:** 2023-06-12

**Authors:** Qiao Cheng, Xiao Huang, Xinying Fan, Jie Sun, Jun Zhang, Qiaoying Tang, Youbin Deng, Xiaojun Bi

**Affiliations:** Department of Medical Ultrasound, Tongji Hospital, Tongji Medical College, Huazhong University of Science and Technology, Wuhan, China

**Keywords:** type 2 diabetes mellitus, myocardial stiffness, intrinsic wave velocity propagation, diastolic function, two-dimensional speck tracking

## Abstract

**Background:**

Diabetes predisposes affected individuals to impaired myocardial perfusion and ischemia, leading to cardiac dysfunction. Increased myocardial stiffness is an independent and significant risk factor in diastolic dysfunction. This study sought to estimate myocardial stiffness in Type 2 diabetes (T2DM) patients using the intrinsic wave velocity propagation (IVP) along the longitudinal wall motion during late diastole and evaluate the value of IVP in assessing cardiac function and structure.

**Methods:**

87 and 53 participants with and without T2DM (control group) were enrolled. Of the 87 T2DM patients (DM group), 43 were complicated with hypertension (DM + H group), and 44 were not (DM-H group). Ultrasound parameters were measured and analyzed, including color M-mode flow propagation velocity, global longitudinal systolic strain (GLS), and IVP.

**Results:**

IVP was higher in the DM group than in the control group (1.62 ± 0.25 m/s and 1.40 ± 0.19 m/s, *P* < 0.001). After stratification for hypertension, IVP in both DM + H (1.71 ± 0.25 m/s) and DM-H (1.53 ± 0.20 m/s) groups were found to be significantly higher than that in the control group (1.40 ± 0.19 m/s); also, the difference of IVP between DM + H and DM-H group reached statistical significance. Moreover, IVP was significantly correlated with flow propagation velocity during early diastole (Pve) (*r* = −0.580, *P* < 0.001), flow propagation velocity during late diastole (Pva) (*r* = 0.271, *P* < 0.001), GLS (*r* = 0.330, *P* < 0.001), interventricular septal thickness at end-diastole (IVSd) (*r* = 0.321, *P* < 0.001), blood glucose (*r* = 0.246, *P* < 0.003), systolic blood pressure (*r* = 0.370, *P* < 0.001) and diastolic blood pressure (*r* = 0.389, *P* < 0.001).

**Conclusions:**

The results indicated the application potential of IVP in assessing the early detection of cardiac function changes noninvasively and sensitively. The correlation with myocardial stiffness warrants further studies to substantiate its potential clinical utility.

## Introduction

Diabetes is a severe global health problem associated with the increased risk of heart failure (1). Diabetes predisposes affected individuals to impaired myocardial perfusion and ischemia, exerting deleterious effects on the myocardium (2). Hyperglycemia facilitates the formation of advanced glycation end products (AGEs), which cross-links with extracellular matrix proteins, leading to increased fibrosis, elevated myocardial stiffness (MS), impaired myocardial relaxation and diastolic dysfunction, and eventually heart failure (3–5). Increased myocardial stiffness is an independent and significant risk factor in diastolic dysfunction (6–8). Thus, early detection of MS is strategic; however, methods assessing MS in diabetes remain poorly studied. Though the gold standard in assessing MS, cardiac catheterization is an invasive method unfit for routine screening (9). The quality of cardiac magnetic resonance imaging with late gadolinium enhancement, on the other hand, is limited by a long scanning time, patient movement, or in those with irregular depth and rate of breathing, as well as arrhythmias despite its application to non-invasive quantification of myocardial fibrosis (10).

Now, the possibility of imaging the heart at very high frame rates allows minute tracking of many wave-like phenomena, such as shear wave elastography technique using acoustic radiation force to mechanically stimulate tissue and monitor the response (11, 12), naturally occurring shear waves generated by aortic and mitral valves closure (13–15).

It was hypothesized that during left ventricular (LV) filling after atrial contraction, the fast traction on the mitral annulus by the atrial contraction generates a wave into the LV, which travels from base to apex with a speed proportional to myocardial elasticity. The propagating wave velocity along longitudinal tissue motion direction is referred to as the Intrinsic Velocity Propagation (IVP) (16, 17). It is generated through the dynamic nature of the heart and allows quantification in all LV segments. It is possible that this wave is related to tissue stiffness and has a radial component. Previous studies (18–20) have demonstrated the feasibility of IVP measurement in normal volunteers and confirmed that IVP was strongly influenced by passive tissue properties. The wave speed was consistent with the pressure-dependent increase in myocardial stiffness, indicating the potential of IVP in evaluating MS.

This work aimed to explore whether there is a relationship between IVP and diastolic dysfunction and discuss the potential of IVP in assessing MS in T2DM patients.

## Materials and methods

### Study population

This pilot study was conducted between April 2019 and March 2021 in Tongji Hospital, the most widely circulating hospital in its region. One hundred forty participants were enrolled, with 87 patients diagnosed as T2DM (DM group) and 53 healthy controls (control group). Among the T2DM patients, 43 were complicated with hypertension (DM + H group), and 44 were exempt from other complications (DM-H group). The diagnosis criteria of T2DM were promulgated by WHO: fasting blood glucose (FPG) ≥ 7.0 mmol/L or 2-hour postprandial blood glucose (2hPG) ≥ 11.1 mmol/L. Patients were excluded from the study if they were complicated with valvular heart disease, congenital heart disease, coronary heart disease, cardiomyopathy, and history of cardiac surgery in case of possible confounding; those with poor echocardiographic image quality were also excluded. The control group was chosen from healthy people undergoing a physical examination, including biochemical routines, electrocardiogram, and echocardiography. They had no history of diabetes, hypertension, cardiovascular disease, or cardiac surgery. All experiments were carried out in accordance with the ethical standards put forward by the Declaration of Helsinki and approved by a local institutional review board. Each participant signed written informed consent.

### Echocardiography examination

Echocardiography was acquired using Vivid E9 commercial scanners (GE Healthcare, Horten, Norway) equipped with an M5S transducer at a frequency of 1.7–3.4 MHz. The rate of conventional two-dimensional ultrasound images is ≥70 frames/s. Three consecutive cardiac cycles are stored in each section. Several conventional echocardiographic parameters were measured, including interventricular septal thickness at end-diastole (IVSd), left ventricular posterior wall thickness at end-diastole (LVPWd), left ventricular internal diameter at end-diastole (LVIDd), left ventricular ejection fraction (LVEF), Mitral E velocity, Mitral A velocity, and Mitral annular septal e' velocity. E/A and E/e' ratios were later calculated.

### Flow propagation velocity examination

The speed scale and scanning speed (100 mm/s) were adjusted to obtain the first-color M-mode aliasing on the apical four-chamber view. Early and late diastolic filling waves on the M-mode map, including flow propagation velocity during early diastole (Vpe) and flow propagation velocity during late diastole (Vpa), were then recorded.

### Global Longitudinal Systolic Strain (GLS)

EchoPac (GE Healthcare) analysis software was applied to calculate GLS offline. The movement trajectory of each point in the cardiac tissue during the cardiac cycle was automatically tracked by two-dimensional speckle tracing in apical long-axis, two-chamber, and four-chamber standard view, respectively. The myocardial deformation of each ventricular wall segment in the region of interest was recorded.

### IVP measurement

**Figure 1:**
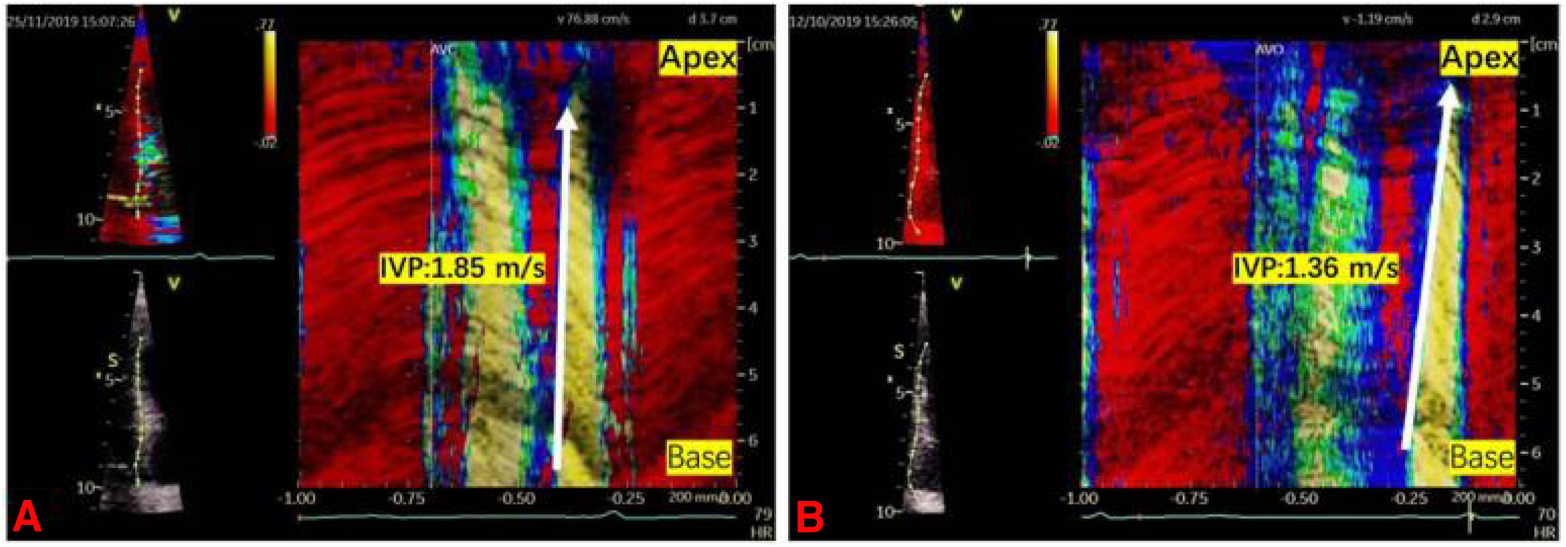
Two examples illustrating the intrinsic wave velocity propagation. IVP was the slope of isovelocity wavefront propagating in time and space during late diastole (the white arrow). (**A**) IVP in a DM-H group patient was 1.85 m/s. (**B**) IVP in a control group participant was 1.36 m/s.

The tissue Doppler frame rate was adjusted to 350–450 frames/s. To minimize the bias caused by the Doppler Angle, the imaging field of view was adjusted carefully to align each left ventricular wall with the incident ultrasound beam. The ultrasound images of each LV wall were stored in apical long-axis, two-chamber, and four-chamber standard views, respectively. Data were recorded and analyzed using the EchoPac workstation (GE Healthcare). Q analysis was first entered, then the left ventricular myocardium was traced using the curved anatomy M type (CAMM) to reconstruct the axial tissue velocity map. The ratio of the tissue velocity map was adjusted to create velocity aliasing; the scanning speed was adjusted to the maximum. The velocity aliasing was used to identify the onset of motion. The wave speed, IVP, was the slope of isovelocity wavefront propagating in time and space during late diastole (Figure 1). The mean IVP measurement was obtained by averaging the wave velocity of six LV walls using the apical four-chamber, two-chamber, and long-axis views.

### Reproducibility analysis

Bland-Altman analysis was applied to test Intra- and inter-observer variability of IVP in 20 randomly selected data sets. Two observers were blind to both results made by each other and the medical history and examination data of each subject.

### Statistical analyses

All statistical parameters were performed using IBM SPSS (Version 24.0. Armonk, NY: IBM Corp). Continuous data were expressed as mean ± SD; categorical data were expressed as percentages. The normality of data was tested using the Shapiro-Wilk test. Between-group comparison of the mean was made by student's t-test; Among-groups comparisons of normally distributed continuous variables was achieved by One-way ANOVA; comparisons of skewedly distributed variables was done by Kruskal Wallis test. When significant at the 0.05 level, pairwise comparisons were performed, and *Post hoc* analysis was achieved through the Bonferroni test. The correlation between IVP and echocardiographic parameters was tested by Pearson correlation analysis. Bland-Altman analysis was used on 20 randomly selected data sets to analyze inter and intra-observer variability and consistency. *P* < 0.05 was considered statistically significant.

## Results

### Primary findings

As was shown in Table 1, patients of the DM + H group were significantly older than those of the DM-H group. There was no difference in T2DM disease duration between DM + H and DM-H groups. However, a significant difference regarding diabetic retinopathy and diabetic nephropathy was observed between the two groups.

**Table 1:** Comparisons of baseline clinical characteristics between the T2DM group and control group.

Variable	Diabetes mellitus (*n* = 87)		Controls (*n* = 53)
	DM + H (*n* = 43)	DM-H (*n* = 44)	
Age (y)	56 ± 1^b^	50 ± 12	54 ± 11
Male (%)	67	61	53
Duration of T2DM (y)	8 ± 7^a^	8 ± 7^a^	__
Blood glucose (mmol/l)	12.57 ± 4.80^a^	14.73 ± 6.08^a^	5.18 ± 0.45
HbA1c (mmol/l)	9.25 ± 1.93^a^	11.48 ± 11.94^a^	5.32 ± 0.43
Systolic blood pressure (mmHg)	149 ± 16^a^^,^^b^	114 ± 11^a^	121 ± 12
Diastolic blood pressure (mmHg)	94 ± 14^a^^,^^b^	77 ± 9	75 ± 9
**Diabetic complications**			
Diabetic retinopathy (%)	44^b^	34	__
Diabetic nephropathy (%)	44^b^	7	__
Diabetic foot (%)	7	7	__

^a^ Significant between DM + H or DM-H group and control group.

^b^ Significant between DM + H and DM-H group.

Overall, the IVP of the DM group was higher than that of the control group (1.62 ± 0.25 m/s and 1.40 ± 0.19 m/s, *P* < 0.001). Such phenomenon was also observed in IVSd (9.85 ± 1.14 mm and 9.23 ± 0.91 mm, *P* = 0.001), Mitral A velocity (84 ± 19 cm/s and 72 ± 21 cm/s, *P* = 0.001), E/A ratio (0.97 ± 0.34 and 1.11 ± 0.35, *P* = 0.016), Mitral annular septal e' velocity (8.45 ± 2.66 cm/s and 9.53 ± 2.28, *P* = 0.015), E/e' ratio(9.68 ± 2.90 and 8.21 ± 2.23, *P* = 0.002), Vpe (54.14 ± 11.20 cm/s and 63.45 ± 10.12 cm/s, *P* < 0.001) and GLS(%)(−17.61 ± 2.20 and −18.60 ± 2.47, *P* = 0.015). After stratification for hypertension (Table 2), The IVP of DM + H group was higher than that of DM-H group and control group (1.71 ± 0.25 m/s, 1.53 ± 0.20 m/s and 1.40 ± 0.19 m/s, *P* < 0.001). Compared with the control group, IVSd (10.21 ± 0.91 mm and 9.23 ± 0.91 mm, *P* < 0.001), Mitral A velocity(90 ± 17 cm/s and 72 ± 21 cm/s, *P* < 0.001), E/e' ratio (10.01 ± 2.92 and 8.21 ± 2.23, *P* = 0.004), Vpa (75.67 ± 17.79 cm/s and 66.94 ± 14.43 cm/s, *P* = 0.027), and GLS(%) (−17.28 ± 2.32 and −18.60 ± 2.47, *P* = 0.018) were significantly higher, while E/A ratio (0.83 ± 0.23 and 1.11 ± 0.35, *P* < 0.001), mitral annular septal e' velocity (7.60 ± 1.90 cm/s and 9.53 ± 2.28 cm/s, *P* = 0.001), and Vpe (51.77 ± 11.94 cm/s and 63.45 ± 10.12 cm/s, *P* < 0.001) were significantly lower in DM + H group patients. Only Vpe in the DM-H group was significantly lower than in the control group (56.45 ± 10.02 cm/s and 63.45 ± 10.12 cm/s, *P* = 0.005). The differences in IVSd, LVIDd, Mitral E velocity, E/A ratio, E/e' ratio, Vpa, and GLS were observed between the DM-H group and control group. However, they did not reach statistically significant.

**Table 2:** Comparisons of echocardiographic parameters between DM + H group, DM-H group and control group.

Variable	Controls	Diabetes mellitus		DM-H		DM + H		P
								DM + H vs. DM-H
	(*n* = 53)	(*n* = 87)	*P* vs. Controls	(*n* = 44)	*P* vs. Controls	(*n* = 43)	*P* vs. Controls	
IVP (m/s)	1.40 ± 0.19	1.62 ± 0.25	<0.001	1.53 ± 0.20	0.013*	1.71 ± 0.25	<0.001*	<0.001*
IVSd (mm)	9.23 ± 0.91	9.85 ± 1.14	0.001	9.50 ± 1.23	0.576	10.21 ± 0.91	<0.001*	0.005*
LVPWd (mm)	9.09 ± 0.88	9.30 ± 1.06	0.240	9.05 ± 1.12	1.000	9.56 ± 0.93	0.067	0.047*
LVIDd (mm)	45.57 ± 3.55	45.10 ± 4.40	0.496	44.86 ± 4.35	1.000	45.35 ± 4.48	1.000	1.000
LVEF (%)	65.17 ± 3.98	65.98 ± 3.65	0.476	65.98 ± 3.50	0.895	65.98 ± 3.85	0.905	1.000
Mitral *E* velocity (cm/s)	76 ± 20	77 ± 19	0.222	82 ± 21	0.443	73 ± 17	1.000	0.105
Mitral *A* velocity (cm/s)	72 ± 21	84 ± 19	0.001	78 ± 19	0.397	90 ± 17	<0.001*	0.017*
E / A ratio	1.11 ± 0.35	0.97 ± 0.34	0.016	1.09 ± 0.37	1.000	0.83 ± 0.23	<0.001*	0.001*
Mitral annular septal *e ‘* velocity (cm/s)	9.53 ± 2.28	8.45 ± 2.66	0.015	9.27 ± 3.03	1.000	7.60 ± 1.90	0.001*	0.005*
E /e ‘ratio	8.21 ± 2.23	9.68 ± 2.90	0.002	9.37 ± 2.87	0.106	10.01 ± 2.92	0.004*	0.793
Vpe (cm/s)	63.45 ± 10.12	54.14 ± 11.20	<0.001	56.45 ± 10.02	0.005*	51.77 ± 11.94	<0.001*	0.128
Vpa (cm/s)	66.94 ± 14.43	70.76 ± 17.51	0.185	65.95 ± 16.01	1.000	75.67 ± 17.79	0.027*	0.016*
GLS (%)	−18.60 ± 2.47	−17.61 ± 2.20	0.015	−17.94 ± 2.04	0.477	−17.28 ± 2.32	0.018*	0.555

GLS, global longitudinal systolic strain; IVP, intrinsic wave velocity propagation of myocardial stretch; IVSd, interventricular septal thickness at end-diastole; LVEF, left ventricular ejection fraction; LVIDd, left ventricular internal diameter at end-diastole; LVPWd, left ventricular posterior wall thickness at end-diastole; Vpa, color M-mode flow propagation velocity during late diastole; Vpe, color M-mode flow propagation velocity during early diastole.

Data are expressed as mean ± SD (median) or as number (percentage).

* *P* < 0.05.

**Figure 2:**
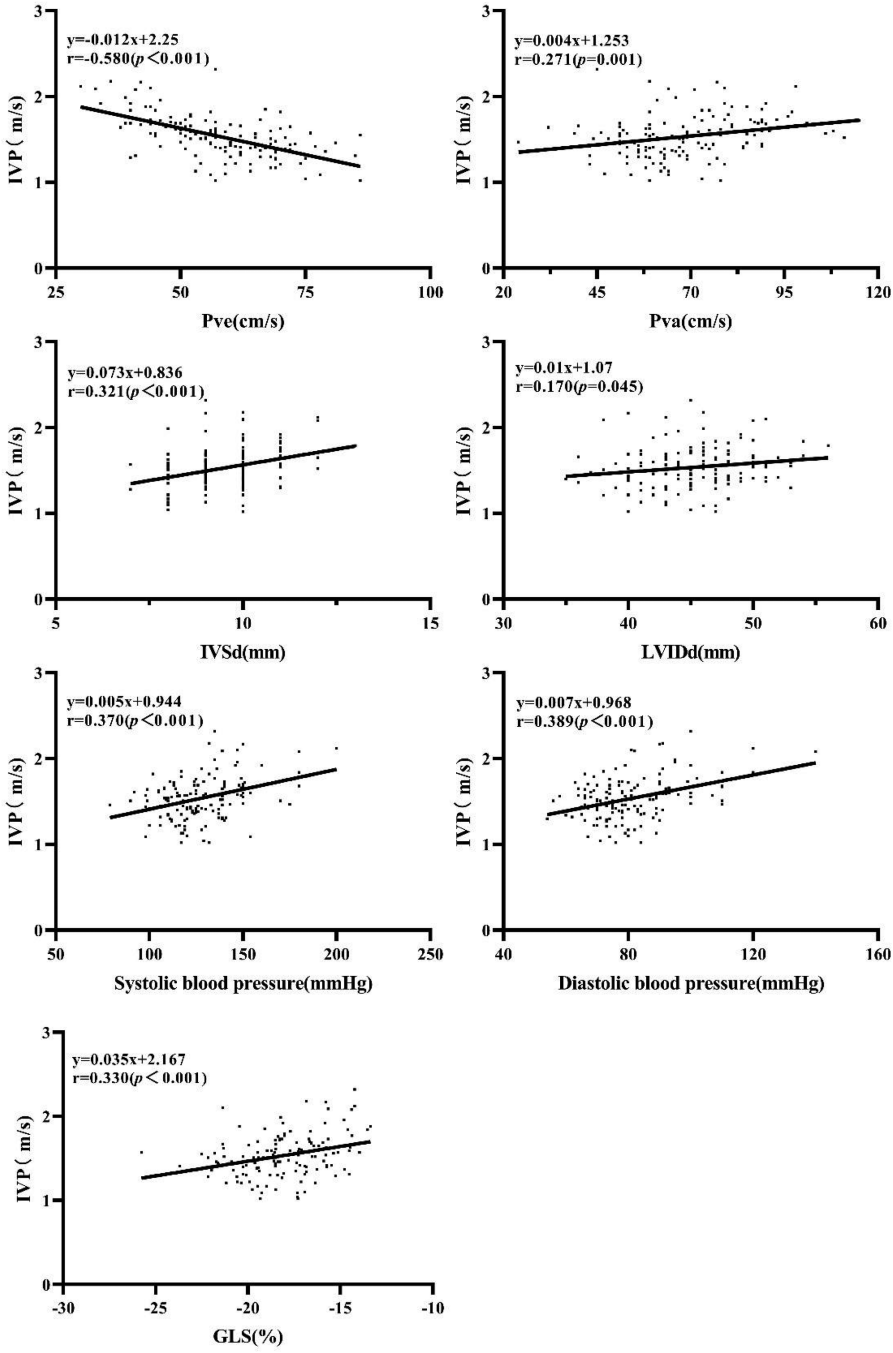
Correlation of IVP with Pve Pva, IVSd, LVIDd, systolic blood pressure, diastolic blood pressure, and GLS.

The results of correlation analyses were as follows (Figure 2): Pve (*r* = −0.580, *P* < 0.001), Pva (*r* = 0.271, *P* < 0.001), IVSd (*r* = 0.321, *P* < 0.001), LVIDd (*r* = 0.170, *P* = 0.045), systolic blood pressure (*r* = 0.370, *P* < 0.001), diastolic blood pressure (*r* = 0.389, *P* < 0.001), and GLS (*r* = 0.330, *P* < 0.001). No significant correlation was found between IVP and HbA1c, blood glucose, LVPWd, LVEF, Mitral E velocity, Mitral A velocity, E/A ratio, e' velocity, and E/e’ ratio.

### Reproducibility and repeatability

**Figure 3:**
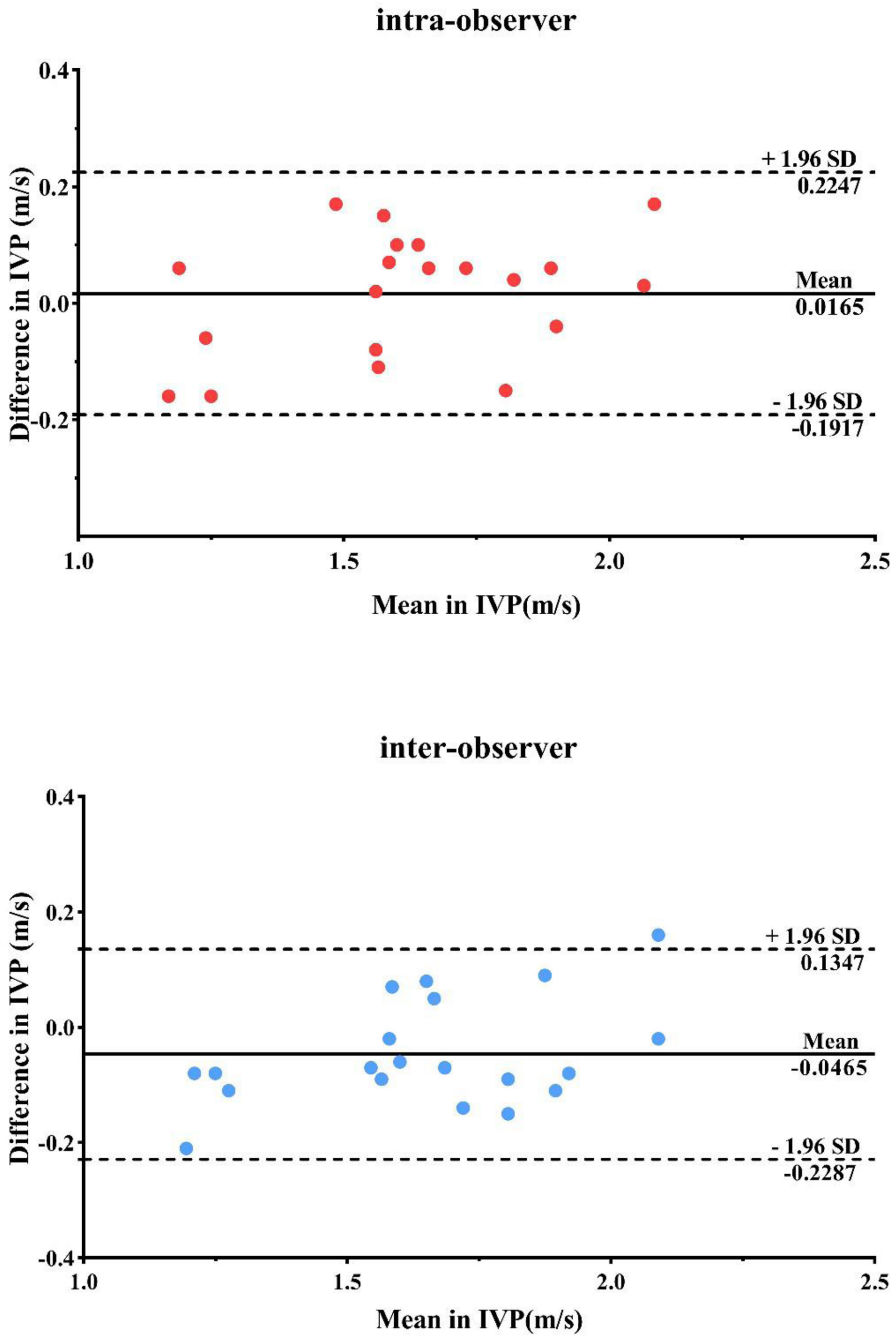
Repeatability and consistency of IVP measurements by bland-Altman analysis within and between observer groups.

The results of the Bland-Altman analysis are illustrated in Figure 3. The intra- and inter-observer reproducibility of IVP was acceptable, with mean signed difference = 0.0165 ± 0.082 m/s and −0.0465 ± 0.1812 m/s, respectively. The intraclass correlation coefficients measured of IVP were 0.924 [95% confidence interval (CI): 0.818–0.969] and 0.941 (95% CI: 0.858–0.976), respectively.

## Discussion

In this work, IVP, the wave propagation of longitudinal myocardial stretch during late diastole, was proposed to evaluate MS in T2DM patients. The main findings from the investigation are as follows: (1) The IVP of the DM + H group was higher than that of the DM-H group and control group; (2) Mitral A velocity, E/e’ ratio, and Vpa were higher in the T2DM patients, while E/A ratio, Mitral annular septal e ‘ velocity, and Vpe were higher in the control group; (3) The association between IVP and part of the above-mentioned conventional ultrasound parameters showcased weak to moderate correlation.

Myocardial relaxation is critical to the diastolic function that allows adequate filling of the ventricles prior to the next cardiac cycle. The early stages of diabetic cardiomyopathy have subtle changes in cardiac function, one of which is left ventricular (LV) diastolic dysfunction that includes impaired early diastolic filling, increased atrial filling, prolonged isovolumic relaxation, and cardiomyocyte disarray and interstitial myocardial fibrosis through histologic examination, the embodiment of altered myocardial stiffness (4, 7).

The generation of IVP is considered at the beginning of ventricular filling after atrial contraction when the base of the LV is stretched, and a pulse-like wave is generated that propagates to the apex along the longitudinal wall motion during late diastole (21, 22) Previous studies in Pislaru et al. series (16, 18, 23) have confirmed that the speed of IVP was strongly influenced by passive tissue properties. The higher the ventricular wall tension, the faster the wave speed. IVP was significantly higher in patients with myocardial amyloidosis characterized by myocardial stiffness than in healthy controls (19). An increase in IVP is associated with incremental diastolic dysfunction, which implies the possibility of a correlation between IVP and myocardial stiffness.

More than two-thirds of T2DM patients are complicated by hypertension (24). Hypertension increases the risk of microvascular and macrovascular complications in diabetic patients (Holman et al. 2008; Kengne et al. 2009). The coexistence of hypertension and diabetes exerts a synergistic adverse effect on impaired subendocardial perfusion that aggravates myocardial oxygen consumption, with resultant abnormal collagen synthesis, altered tissue elasticity, and elevated myocardial stiffness (25, 26). After stratification for hypertension, the statistical difference of several morphologic and hemodynamic indicators between DM + H, DM-H, and the control group was likely associated with the combined effects of elevated filling pressures, wall stress, and LV afterload, which indicate increased diastolic dysfunction and myocardial stiffness in the case group. Despite the values of IVP being similar between controls and cases and the moderate correlation with indicators, the increasing trend should not be overlooked; besides, cardiac function cannot be embodied by a single parameter. In this study, the IVP value tends to be higher as myocardial condition deteriorates. IVP exhibited a positive association with systolic blood pressure, diastolic blood pressure, IVSd, and LVIDd. It is in line with our previous results (20), which disclosed that IVP was higher in hypertensive patients than in controls and was closely related to LVIDd and LVPWd.

The blood flow from the LA to the LV through the mitral valve can be embodied as the wave; the speed produced under the pressure gradient in the ventricle is referred to as the flow propagation velocity (FPV). FPV is a recognized indicator of changes in myocardial mechanical properties. Reduced flow propagation during early diastole is commonly seen in patients with impaired LV relaxation (27, 28). When LA filling is impaired, the isovolumic relaxation time is prolonged, accompanied by decreased Vpe. As it develops, the atrial contractions increased compensatively to overcome the early diastolic filling impairment caused by myocardial stiffness; simultaneously, Vpa increases. In the current study, there was a negative correlation between IVP and Vpe and a positive correlation between Vpa, which theoretically supported the potential utility of this new measurement in predicting myocardial stiffness. Nevertheless, flow propagation measurements are also angle-dependent, the volume of blood in early flow propagation (Vpe) influences late flow propagation, and the Vpa slope is uncertain (29), which may partly explain the insignificant association between the DM-H group and the control group in our study. Future research with a larger sample size and a broader spectrum of the population is warranted to address this issue.

E/e' ratio is one of the well-recognized parameters in evaluating LV filling pressure diastolic function (30). In this study, the E/e’ ratio was 9.68 ± 2.90 in the DM group, between 9 and 14, which indicated intermediate LV filling pressures; also, there was an insignificant correlation between IVP and E/e ‘ ratio. Diastolic function is a complex process and cannot be evaluated by a single parameter. Despite the negative results, diastolic dysfunction cannot be ruled out due to its low sensitivity (or specificity). GLS is highly sensitive and specific in detecting LV dysfunction (31, 32). Weakened myocardial elasticity and increased myocardial stiffness are associated with decreased GLS, which is consistent with the alteration tendency of IVP in myocardial dysfunction disclosed in this study. It might serve as a complementary method in analyzing myocardial dysfunction.

Here, this work explored the application prospect of IVP in assessing LV diastolic dysfunction in T2DM patients. The results implied that IVP might be potential to evaluate diastolic dysfunction and reflect the change in myocardial stiffness. Different from the generation of acoustic radiation force pulse in shear wave elastography, this novel ultrasound technique detects the wave velocity generated by myocardial stretch from the base to the apex level through high frame rate Doppler ultrasound imaging, which means the external stimulation is not required (33, 34). The convenience of IVP measurement makes it available for research. This novel index might be a potentially highly sensitive and harmless indicator in evaluating early cardiac function variations in some cardiovascular conditions, such as T2DM patients. With technological advancement, such as higher frame rates and velocity range, more accurate measurements are available, which may be helpful in clinical applications.

## Study limitations

Several limitations deserve to be addressed: The data quality of IVP is affected by time resolution and low frame frequency. The application of this technique is currently unsuitable for patients with arrhythmias or tachycardia. Limited by experimental conditions, in this pilot study, the association of IVP with myocardial stiffness was relatively subjective, for there is currently no direct reference standard. Limited by the small sample size, one possibility is that the group variability may be due to inter/intra-observer variability. However, the trend of IVP value changes could not be ignored and was worthy of further exploration. Myocardial stiffness also can be acutely modulated by proteins such as titin, including post-translational modifications (PTMs, such as acetylation, oxidation, and phosphorylation) and isoform switch (35). The atrial contraction in end-diastole leads to a rapid increase in LV strain, which might result in a rapid increase in momentary stiffness in T2DM. More clinical studies with larger sample sizes and combined indicators are warranted further investigation to prove the clinical value of IVP.

## Conclusions

The study showed that IVP was higher in T2DM patients when compared to the control group. This novel index might be a promising echocardiographic parameter in assessing early change in cardiac function and structure, implying the possibility of evaluating myocardial stiffness directly and noninvasively. Further clinical studies with larger sample sizes are warranted to explore the clinically relevant.

## Data Availability

The raw data supporting the conclusions of this article will be made available by the authors, without undue reservation.
